# A detailed phenotypic assessment of individuals affected by *MFRP*-related oculopathy

**Published:** 2010-03-26

**Authors:** Rajarshi Mukhopadhyay, Panagiotis I. Sergouniotis, Donna S. Mackay, Alexander C. Day, Genevieve Wright, Sophie Devery, Bart P. Leroy, Anthony G. Robson, Graham E. Holder, Zheng Li, Andrew R. Webster

**Affiliations:** 1 Department of Genetics, Institute of Ophthalmology, University College London, UK; 2 Moorfields Eye Hospital NHS Foundation Trust, London, UK; 3 Department of Ophthalmology & Centre for Medical Genetics, Ghent University Hospital & Ghent University, Ghent, Belgium

## Abstract

**Purpose:**

To determine the spectrum of mutations and phenotypic variability within patients with mutations in membrane-type frizzled related protein gene (*MFRP*).

**Methods:**

Individuals were initially ascertained based on a phenotype similar to that previously published in association with *MFRP* mutations. Affected patients underwent a full ophthalmic examination (best-corrected visual acuity, slit-lamp examination, applanation tonometry, and fundoscopy), color fundus photography, optical coherence tomography, autofluorescence imaging, and electrophysiology. *MFRP* was identified by a genome-wide scan in the fourth-largest autozygous region in one consanguineous family. Sanger sequencing of all the exons and intron-exon boundaries of *MFRP* was undertaken in the affected individuals.

**Results:**

Seven affected individuals from four families were identified as having mutations in *MFRP*. Patients from two families were homozygous for mutations already previously described (c.1143_1144 insC and c.492 delC), while those from the other two were compound heterozygous for mutations (c.201G>A and c.491_492 insT, and c.492 delC, and c.1622_1625 delTCTG), three of which were novel. There was considerable phenotypic variability within and among families. Autofluorescence imaging revealed the central macula to be relatively well preserved. Foveal cysts and optic nerve head drusen were present in two of the four families. Electrophysiology results showed rod-cone dystrophy with mild to moderate reduction in macular function in all affected members.

**Conclusions:**

We report three novel *MFRP* mutations and expand the phenotypic data available on patients with *MFRP* mutations.

## Introduction

Recessive mutations in the membrane-type frizzled-related protein (*MFRP,* OMIM *606227) gene have recently been reported to cause human ocular disease. However, two different phenotypes have been described. Several research groups have described families with affected members having high hyperopia, shallow anterior chambers, optic nerve head drusen, optic nerve head pallor, pigment clumping, and bone-spicule pigmentation, in both the midperiphery and periphery, and minimal vascular attenuation [1-3]. A further publication reported three families with recessively inherited nanophthalmos recessively inherited nanophthalmos as a consequence of upon mutation in *MFRP* [4]. Interestingly, the retinas of the affected patients showed patchy hypopigmentation without any evidence of retinal dystrophy.

The 13-exon *MFRP* gene was cloned and described in 2001 [5], and is located on chromosome 11q23. It encodes a transmembrane protein with 579 amino acid residues that is expressed predominantly in the retinal pigment epithelium and ciliary epithelium of the eye, with a weak expression in the brain [5]. The exact role of *MFRP* has yet to be established. It has a frizzled-type cysteine-rich domain, which is the binding motif for Wingless (Wg), a member of the Wnt family of proteins. Several processes, such as control of gene expression, cell adhesion, planar polarity, proliferation, and apoptosis involve Wnt signaling. It is suggested that *MFRP* is involved in the Frizzled/Wnt signaling pathway. Besides having a cysteine-rich domain at the C-terminus, the *MFRP* protein contains an N-terminal cytoplasmic region, a transmembrane domain, and an extracellular region with tandem repeats of two cubilin domains and a low-density lipoprotein receptor-related sequence [5].

There is a murine model for the *Mfrp* mutation: the retinal degeneration 6 (*rd6*) mouse. The retinal phenotype is characterized by small, evenly spaced white dots throughout the retina, together with a slowly progressive retinal degeneration of both rods and cones, evidenced by electroretinography [6]. Later, using the same mouse model, it was reported that *Mfrp* is expressed as a dicistronic transcript with another retinal dystrophy causing gene *C1qtnf5* [7]. *C1qtnf5* and *Mfrp* have been shown to co-localize to the same tissues, and consequently a functional relationship between the two proteins was inferred [8].

The present report extends our knowledge of the ocular phenotype. It describes a series of four families with mutations in *MFRP*, the largest series reported to date. Three novel mutations are reported.

## Methods

Seven patients from four families were identified, based on a phenotype similar one described in previous reports of *MFRP*-related disease [1-3]. This study adhered to the tenets of the Declaration of Helsinki and was approved by the local ethics committee.

### Clinical investigations

Patients underwent full ophthalmic examination (best-corrected visual acuity, slit-lamp examination, applanation tonometry, and fundoscopy) at Moorfields Eye Hospital (London, UK). Color fundus photography was performed using a Topcon TRC 50IA retinal camera (Topcon Corporation, Tokyo, Japan), fundus autofluorescence imaging using a confocal scanning laser ophthalmoscope (Zeiss Prototype; Carl Zeiss Inc., Oberkochen, Germany) and optical coherence tomography (OCT) using a SPECTRALIS® Spectral-domain OCT (Heidelberg Engineering, Heidelberg, Germany) and a STRATUSOCT Model 3000 scanner (Zeiss Humphrey Instruments, Dublin, CA). Color vision was tested using Hardy-Rand-Rittler plates (American Optical Co., New York, NY) and/or the Ishihara pseudoisochromatic plates (Kanehara Trading Inc., Tokyo, Japan). To seek the role of *MFRP* in determining the axial length of the eye, the refractive status of the obligate carriers was evaluated.

Electrophysiological assessment was performed in four affected patients. Full-field electroretinograms (ERGs) and pattern ERGs incorporated the methods recommended by ISCEV [9,10]. Dark adapted ERGs were recorded following stimulation with white flashes of intensity between 0.01 and 11.5 cd.s.m^−2^. An additional red-flash ERG from healthy individuals who had both a cone and a later rod component, were recorded, to compare the normal function of dark-adapted cone and rod systems to that of affected individuals. Generalized photopic cone system function was assessed after 10 min of light adaptation by recording 30 Hz flicker and single flash ERGs (3.0 cd.s.m^−2^ flash, 30 cd.m^−2^ background illumination). ON-OFF ERGs used an orange stimulus (560 cd.m^−2^, duration 200 ms) superimposed on a green background (150 cd.m^−2^). Short wavelength flash ERGs used a blue stimulus (445 nm, 80 cd/m^2^) on an orange background (620 nm, 560 cd/m^2^).

### Molecular genetics

Genomic DNA was extracted from peripheral blood leukocytes of the donated blood samples (Nucleon BACC2 kit; GE Healthcare, Buckinghamshire, UK) after obtaining informed consent. DNA samples from family 1 (consanguineous) were genotyped for 58,614 SNPs using a Genechip Human Mapping 50K Xba array (Affymetrix, Santa Clara, CA). Samples were prepared by using Affymetrix GeneChip® Mapping 50K Xba Assay Kit as per the manufacturer’s instructions (Affymetrix). Briefly, 250 ng of genomic DNA was digested with XbaI (New England Biolabs, Ipswich, MA) for 2 h at 37 °C. It was ligated with an XbaI adaptor by using T4 DNA ligase. The ligation reaction was diluted to 1:4 (v/v) with molecular-grade water (Sigma-Aldrich Corporation, St. Louis, MO) to 100 μl. Ten μl of the diluted ligation mix was used to set up the following. Fragment selection was done by PCR in triplicates. The consolidated PCR products were purified using 96-well plates (Qiagen Gmbh, Hilden, Germany). The concentration of PCR product was quantified by using ND-1000 Nanodrop spectrometry (Thermo Fisher Scientific, Waltham, MA). Purified PCR product (90 μg) was fragmented with 0.25 units of fragmentation reagent and labeled with labeling reagent. Of the labeled product, 70 μl was mixed with 190 μl of hybridization reagent, and denatured at 99 °C for 10 min. Finally, this denatured hybridization cocktail was injected into Affymetrix Human Mapping StyI chips (Affymetrix) and incubated at 49 °C for 16 h, followed by automated washing and staining in a Fluidics Station 450 (Affymetrix). Scanning was done with a GeneChip® Scanner 3000 7G (Affymetrix).

All chip data were imported into the GeneChip® Operating Software (Affymetrix) software platform and genotype data were extracted. The pedigree was consistent with the propagation of a single mutant allele from a recent ancestor, such that both affected probands were homozygous for this allele. A macro was written using the Visual Basic program in Microsoft Excel (Microsoft, Redmond, WA) to detect genomic regions obeying this rule.

The *MFRP* gene was screened for presumed disease-causing mutations. Primers were designed to amplify the coding region and intron-exon boundaries of the 13 published exons (NM_031433.2; Table 1). All PCRs were performed in a 25 μl volume containing 1X reaction buffer and 0.1X of Enhancer (Molzym, Bremen, DE), 200 μm each of dNTP, 10 pMol each forward and reverse primer, 200 ng to 1 μg DNA, and 1U Taq polymerase (MolTaq, Molzym). After the denaturation step at 94 °C for 5 min, the amplification was for 34 cycles at 94 °C for 30 s. Annealing was performed at 60 °C for 30 s, and then at 72 °C for 45 s. The final extension was at 72 °C for 5 min.

**Table 1:** Primers used for polymerase chain reaction of *MFRP*.

**Amplimer**	**Forward primer (5′–3′)**	**Reverse Primer (5′–3′)**
1 (Exon 1–Exon 3)	CCACTCTACCAAGGACAGCCCAAGAA	CATTCCAAAGCCCTCGTTTCAA
2 (Exon 4–Exon 5)	CTCTGAACGCCACCCTCCATCTT	GCCACTCCCTGATTCTGCTCTTTAGATA
3 (Exon 6–Exon 8)	CTCACCCCCAACCTGGCTTCTT	GTGTCTACCATGCCATTCCCATTA
4 (Exon 9)	GAGACAGGAGAAGGGGCCATGAATT	CCGGCCTGGAGTAGCAGAAGAAAA
5 (Exon 10–Exon 11)	CCCTAACCCTGTGTCTTCCATCACCTTT	CCGACTGTCTTCCAGGACTCTGTGAA
6 (Exon 12–Exon 13)	CCACCCCCATTGGACCCATGTA	CCTGCTGATGCTCCTTCCTTTGTT

Amplicons were run on a 2% (w/v) low-melting-temperature agarose gel in 1X Tris-acetate- EDTA (TAE) buffer to check for the quality and specificity of the PCR reaction. The products were cleaned, using multiscreen PCR filter plates (Catalogue no LSKMPCR10, Millipore, Billerica, MA) before sequencing. Bi-directional Sanger sequencing of purified PCR products was performed using BigDye Terminator sequencing Cycle Sequencing Ready Reaction kit version 1.1 (Applied Biosystems Inc., Foster City, CA) and separating sequenced fragments on a 3730 genetic analyzer (Applied Biosystems, Warrington, UK). Lasergene version 8.02 sequencing analysis software (DNASTAR Inc., Madison, WI) was used to compare electropherograms with those from control samples.

## Results

### Clinical phenotype

**Figure 1:**
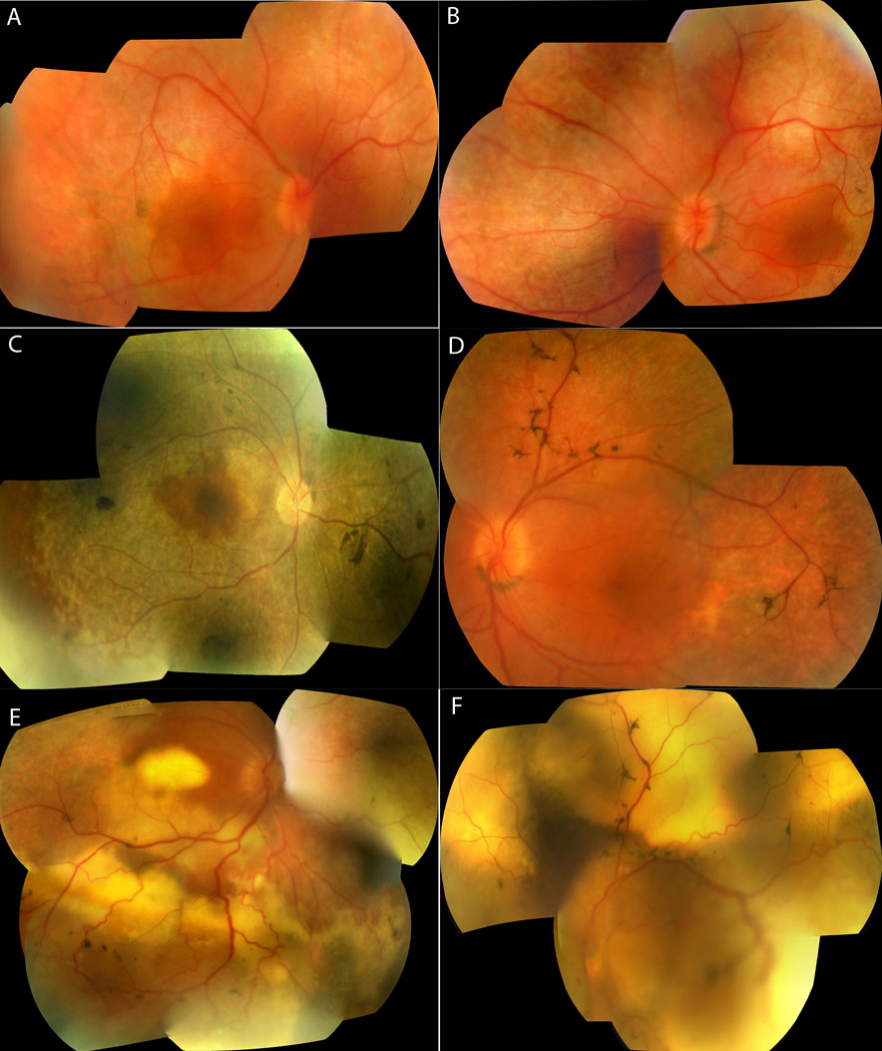
Color fundus photographs of affected patients with membrane-type frizzled-related protein gene related oculopathy. These show the right (**A**) and left (**B**) fundus photographs of subject III-1 of family 4, the right fundus photograph of individual II-1 of family 1 (**C**), and the left fundus photograph of patient III-3 of family 2 (**D**); all show an ellipsoid area of macula with normal reflex. Fundus photographs show the right (**E**) and left (**F**) eye of individual III-3 of family 2 after developing serous retinal detachments.

**Figure 2:**
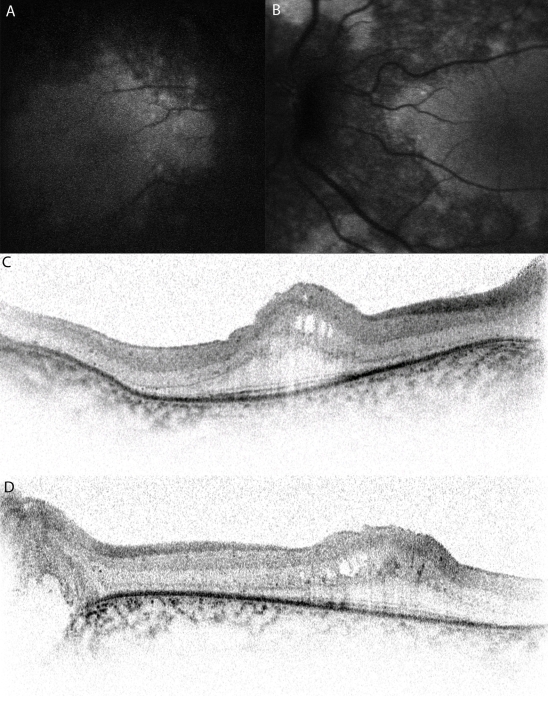
Autofluorescence images and high-resolution spectral domain optical coherence tomograms of affected patients with membrane-type frizzled-related protein, and related oculopathy. Autofluorescence images of the right (**A**) and left (**B**) eyes of posterior pole of individual III-1 of family 4 show relative preservation of the central macula. Optical coherence tomograms of the right (**C**) and left (**D**) fovea of individual II-1 of family 3 show areas of low reflectivity, which are due to cysts and relative thickening of the retina in the foveal region.

The clinical data of the seven patients are detailed in Table 2, Figure 1, and Figure 2. The age of presentation ranged between 34 and 58 years, although all patients noticed visual acuity problems from early childhood, due to hypermetropia. Four of seven patients complained of night blindness at birth or early childhood, while two did not report night blindness. Only one of seven patients was photophobic.

**Table 2:** Clinical features of the affected patients in families 1–4.

**Individual**	**Age in years**	**Gender**	**Age on onset of night blindness**	**Refractive error (spherical equivalent)**	**Best corrected visual acuity**	**Foveal cystic spaces**	**Optic nerve head drusen**	**Cataract**
**Family 1**								
II–1	58	Female	Early childhood – first decade	+15.00 D right +16.00 D left	3/60 both eyes	Absent	Absent	Bilateral cataract surgery
II–2	53	Female	Since birth	+19.00 D right +20.00 left	6/60 both eyes	Absent	Absent	Absent
II–5	46	Female	Since birth	+13.00 D right +14.00 D left	6/18 both eyes	Absent	Absent	Absent
**Family 2**								
III–2	50	Female	Never	+19.00 D right +20.00 D left	6/9 right 6/12 left			Absent
III–3	47	Female	18 years	+18.00 D right +18.50 D left	NPL right HM left	Present	Absent	Bilateral cataract surgery
**Family 3**								
II–1	42	Male	Early childhood (before 5 years of age)	+30.00 D right +27.75 D left	6/60 both eyes	Present	Present	Absent
**Family 4**								
III–1	34	Female	Never	+17.75 D right +18.00 D left	6/60 both eyes	Present	Absent	Absent

Abbreviations: D represents Diopters; NPL represents No perception of light; HM represents Hand movements.

Best-corrected visual acuity of the better eye was less than or equal to 6/60 in five patients; only one patient had visual acuity better than 6/12. One patient (III-3 of family 2) lost her central vision at 42 years of age, due to bilateral progressive serous retinal detachments.

All seven patients were highly hypermetropic, with spherical equivalents ranging between +13.00 diopters (D) and +30.00 D. The refractive status of most of the obligate carriers in all four families was also examined. The two children of individuals III-2 and III-3 of family 2, the three children of II-1, and the only child of II-5 of family 1 were emmetropic. The children of II-1 of family 3 were not examined. None of the parents of the affected subjects in families 1, 2, or 3 needed refractive correction, except for presbyopia. However, one parent (II-14) of the affected siblings in family 2 was hypermetropic (spherical equivalent +3.00 D for both eyes). Longitudinal estimation of the refractive status of subject II-5 of family 1 for nearly three decades showed that the visual acuity and hypermetropia remained stable (data not shown). None of the patients had nystagmus. All three patients tested for color vision failed to read the test plate.

Retinal examination revealed extensive mid-peripheral retinal pigment epithelial changes, with relative preservation of central macular reflex in all patients. There was arteriolar attenuation and intraretinal pigmentation (Figure 1). One patient (III-3 of family 2) developed extensive multiple serous detachments. Optic nerve head drusen were present in only one (II-1 of family 3) of the seven patients. Autofluorescence imaging of one affected subject (III-1 of family 4) showed preservation of a normal autofluorescence in an elliptical region at the central macula, with hypo-autofluorescence observed in the peripheral macula (Figure 2). OCT examination of three patients (III-3 of family 2, II-1 of family 3, and III-1 of family 4) revealed thickening of the inner layers and cystic spaces at the fovea, with relative preservation of the photoreceptor-retinal pigment epithelium complex. There was thickening of the outer retinal tissue, over and above the cysts, in at least one patient (II-1 of family 3). The foveal thickness varied from 502 μm to 673 μm.

### Electrophysiology

**Figure 3:**
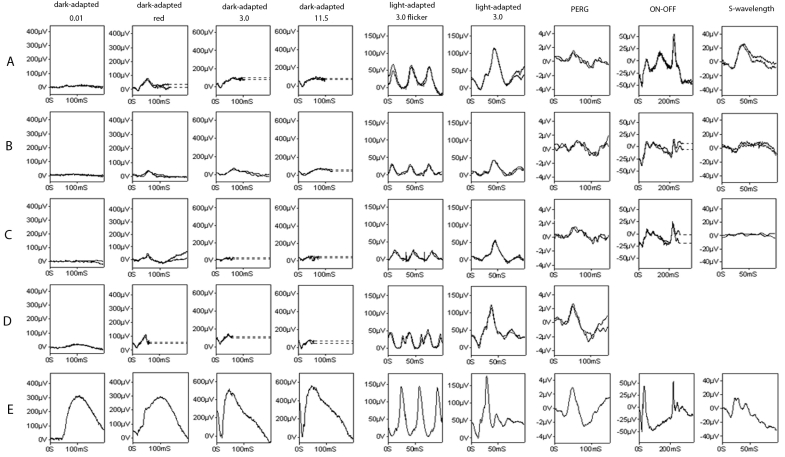
Electroretinograms of the patients affected with membrane-type frizzled-related protein, and related oculopathy. Electroretinograms (ERGs) and pattern ERGs of subject III-1 of family 4 (**A**), II-1 of family 1 (**B**), and III-3 of family 2 (**C**) show severely decreased rod photoreceptor function and moderately affected cone photoreceptor function. There is a moderate decrease in macular function, as shown by the pattern ERGs. Though similar ERG findings are seen in individual III-2 of family 2 (**D**), the pattern ERG is relatively well preserved, compared to normal case (**E**).

Bright flash (Figure 3) dark-adapted ERGs showed marked a-wave reduction, in keeping with loss of rod photoreceptor function. Scotopic red flash ERGs had an undetectable rod component and a severely delayed cone component. Photopic flicker ERGs were severely delayed and showed mild to moderate reduction in all cases, in keeping with generalized retinal cone system dysfunction. There was an abnormal flicker ERG waveform in patient III-3 of family 2. Single flash cone ERGs were delayed in all four cases, in addition to mildly subnormal amplitudes in patient II-1 of family 1 and patient III-2 of family 2. Both the On b-waves and Off d-waves were delayed in three out three subjects. Short wavelength flash ERGs had a subnormal S-cone component in three out three subjects (II-1 of family 1, III-3 of family 2, and III-1 of family 4). Pattern ERG P50 was mildly subnormal in three out four subjects, and was within normal limits in the fourth patient. The phenotype is thus of a rod-cone dystrophy, with macular involvement in three patients (III-1 of family 4, II-1 of family 1, and III-3 of family 2), but lacking macular involvement in the fourth (III-2 of family 2).Though there are limited follow-up data available, ERGs recorded in subject III-3 of family 2, aged 41 years, did not significantly differ from those recorded 3 years earlier (data not shown).

### Molecular genetics

**Figure 4:**
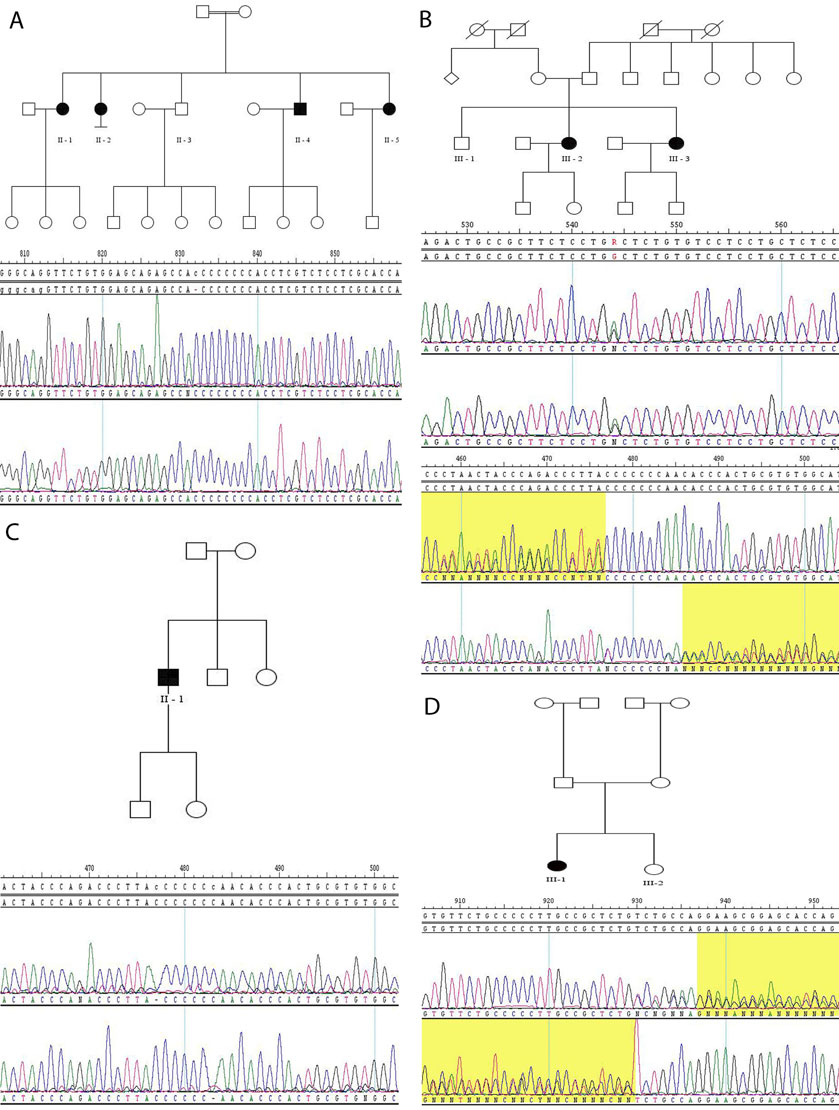
Pedigrees and electropherograms of families 1–4. The dark symbols in the pedigrees represent affected individuals. The electropherograms show bi-directional sequencing of the homozygous c.1143_1144 insC mutation in family 1 (**A**), compound heterozygous c.201 G>A and c. 491_492 insT mutations in family 2 (**B**), homozygous c.492 delC mutation in family 3 (**C**), and heterozygous c.1622_1625 delTCTG mutation in family 4 (**D**). The heterozygous c.492 delC mutation in family 4 is not shown.

The pedigrees of each of the families are illustrated in Figure 4. Family 1 had three affected individuals born out of a first-cousin marriage. The genome-wide scan revealed five large regions of autozygosity. The two largest regions were on chromosome 2, the third-largest on chromosome 17, the fourth-largest on chromosome 11, and the smallest on chromosome 4. The fourth region consisted of 12.8 Mb, which included 187 contiguous SNPs. Upon interrogating the Ensembl database, one obvious candidate found was the *MFRP* gene. Sanger sequencing of *MFRP* revealed homozygous mutation p.His384Pro fs8X (Table 3), which was segregated with disease among the affected members of the family.

**Table 3:** Mutations found in families 1–4.

**Family**	**Mutations**	**Protein**	**Reference**
1	c.1143_1144 insC (homo)	p.His384Pro fs8X	[4]
2	c.201G>A (het) and c.491_492 insT (het)	p.Trp67X and p.Asn167Gln fs34X	Novel
3	c.492 delC (homo)	p.Asn167Thr fs25X	[4]
4	c.492 delC (het) and c.1622_1625 delTCTG (het)	p.Asn167Thr fs25X and p.Val541Ala fs188X	[4] and Novel

The compound heterozygous mutations found in family 2 and one of the compound heterozygous mutations in family 4 are novel.

**Figure 5:**
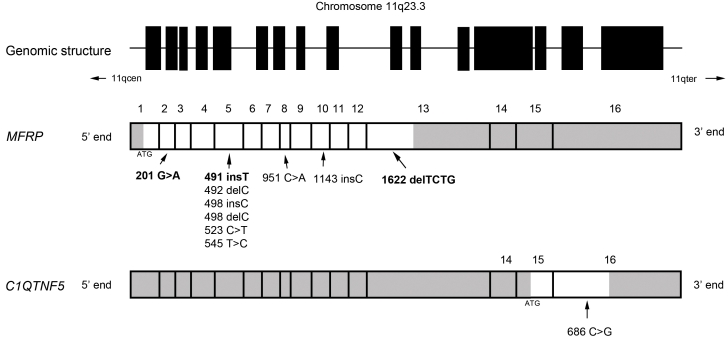
Structure of membrane-type frizzled-related protein with position of mutations. The upper panel represents the genomic structure of the dicistronic *MFRP-C1QTNF5* with the exons demonstrated as filled boxes and the intervening introns as a line. The small arrows show the positions of the centromere (11qcen) and the terminal end (11qter) of the large arm of Chromosome 11. In the middle panel, the membrane-type frizzled-related protein (*MFRP*) coding region is shown with the untranslated regions colored gray, with ATG representing the start codon and with the exons numbered from 5′ end to 3′ end. The positions of all the published mutations are shown; the novel mutations are in bold. The bottom panel shows the coding region and the only published mutation of *C1QTNF5* in a similar fashion. All the exons are drawn to scale.

Sequencing of *MFRP* in affected members of three other families with similar phenotype, families 2, 3, and 4, revealed more mutations (Table 3 and Figure 5). Affected individuals in family 2 showed two novel compound heterozygous mutations (p.Trp67X and p.Asn167Gln fs34X), while the proband in family 3 was homozygous for a known mutation (p.Asn167Thr fs25X) [4]. The patient in family 4 was compound heterozygous for one known (p.Asn167Thr fs25X) [4] and one novel mutation (p.Val541Ala fs188X). All mutations led to a premature stop codon.

## Discussion

This study describes the detailed phenotype of patients having a retinal dystrophy as a consequence of homozygous or compound heterozygous mutations in *MFRP*. This report represents the largest series described to date, and greatly expands our knowledge of both clinical and electrophysiological phenotypes and genotypes by reporting three novel mutations.

All the affected subjects noticed visual difficulties in childhood, but the onset of night blindness was variable. Patients from family 1 and 3 reported early-onset night blindness, while two subjects (III-2 of family 2 and III-1 of family 4) did not have problems in dim light. Only one patient, III-3 of family 2, manifested night blindness at 18 years of age, which was similar to the family described by Ayala-Ramirez et al., [1] where the proband complained of night blindness at 24 years of age. The poor visual acuity can be explained by fovea being affected by the disorder early in the disease, or by foveal cysts. Because the patients were high hypermetropes, amblyopia could also have contributed to reduction in visual acuity.

The refractive analysis of obligate carriers revealed that only one out of 15 obligate carriers was hypermetropic. Thus, haploinsufficiency for this gene does not cause a significant departure from optimal emmetropization, at least in this modest sample. It is therefore unlikely that more subtle differences in expression or function of the *MFRP* gene contribute to refractive error variability in the normal population. This adds further evidence to the previously published observation that *MFRP* does not play a significant role in physiologic hypermetropia [11]. The longitudinal refractive data was stable, which suggests that the nanophthalmic component of this disorder progresses slowly after puberty, if at all. Homozygosity or compound heterozygosity for *MRFP* mutations does seem to be associated with considerable reduction of axial length, as proposed by others [4,12].

There was considerable phenotypic variability within and between the families. For example, optic nerve head drusen were present in one out of four families. The most striking discordance was found between the two affected sisters in family 2. One of the sisters (III-3) developed multiple elevated serous retinal detachments, which had been operated upon multiple times, albeit without improvement of visual acuity. She complained of night blindness from 18 years of age, had poor visual acuity, and had a decreased pattern ERG. However, her older sister (III-2), who had no serous retinal detachment, had an unaffected pattern ERG, and had retained good central vision and visual fields. She did not yet have any night blindness. The serous detachments could have been due to a nanophthalmos component of the phenotype. Although we did not have data on the scleral thickness of the affected subject, ultrasound examination of subject II-1 of family 1 showed the sclera to be of normal thickness.

The fundus examination of most of the affected subjects showed remarkable similarity in the preservation of the posterior pole reflex, which corresponded to an ellipsoid preserved region of autofluorescence (Figure 1 and Figure 2). Though not discussed in previous reports, the fundus photographs of patients from two different groups have a shown similar pattern of preserved posterior pole reflex [1,2]. There are no previous data on autofluorescence on affected subjects with *MFRP* oculopathy.

Only half of the patients showed the presence of foveal cysts upon clinical examination or OCT (Figure 2). The OCT data did not conclusively determine or discount foveal schisis. This is different from the data published by other groups, in which foveal schisis was the norm [1-3].

Electrophysiologically, all the ERGs in our patients showed a rod-cone pattern of dysfunction, with severe rod system involvement. Although this is different from the families described by Sundin et al. [4], whose affected patients had no retinal degeneration, similar findings have previously been described [1-3]. In our cases, the scotopic bright flash ERG a- and b-waves were subnormal. They resembled the delayed cone component of the red flash ERG. This was in keeping with a dark-adapted cone system origin, exposed in the presence of severe rod photoreceptor dysfunction. Pattern ERGs have not been described previously for this condition, and ours were consistent with either relative or complete sparing of central macular function. Our ERG findings were not pathognomonic for *MFRP* mutation, but were consistent across the individuals tested, and may help identify other candidates for *MFRP* screening. The ERG phenotype is similar to that observed in *rd6* mice, allowing for the differences in retinal structure between man and mouse. It may be at least partially explained by impaired retinal pigment epithelium phagocytosis, and by faulty development of photoreceptor outer segments [13].

Similar to the mutations identified in our families, all but one of the previously described mutations were either frameshift or nonsense [1-4]. The only missense change found was p.I182P in exon 5 in one family [4]. All but one of the mutations reported in this series would be expected to cause nonsense-mediated decay [14], because they would lead to a premature truncation codon before the 5′-most splice site of the gene. However, since the mRNA is dicistronic, containing the exons of the gene *C1QTNF5* [7], nonsense-mediated decay would abrogate expression of both genes. Immunohistochemistry in the mouse model has shown that in-frame deletion of *Mfrp* failed to produce a mutant *Mfrp* protein, although the expression of *C1qtnf5* was unaffected [13]. One parsimonious explanation for the effect of these mutations is that the mutant allele is translated, but the protein is degraded post-translationally, leading to a null phenotype. An alternative is that the phenotype is the result of non-expression of both *MFRP* and *C1QTNF5*, although we feel that this is unlikely, given the absence of any signs that resemble the ocular disorder, late-onset retinal dystrophy, caused by mutation of *C1QTNF5*. The p.Val541Ala fs188X mutation is an exception and would be expected to cause an elongation of the protein with a new C-terminus. There was no evidence of association between the type of mutation and the severity of the phenotype. The three novel mutations that we report in this paper are either nonsense mutations (p.Trp67X) or frameshifts (p.Asn167Gln fs34X and p.Val541Ala fs188X). Because of the profound effect that these have in the protein, we have not screened a control panel for these specific variants.

To conclude, we have expanded and refined the phenotypic description of patients with the rod-cone dystrophy associated with mutations in the *MFRP* gene. The clinical and electrophysiological features described here should alert us to the possibility of *MFRP* mutation as a potential cause of similar diseases in other patients.
